# Evaluation of Clinical Assessment in Predicting Complicated Acute Diverticulitis

**DOI:** 10.7759/cureus.34709

**Published:** 2023-02-06

**Authors:** Hassan Al-Saadi, Haider Abdulrasool, Elizabeth Murphy

**Affiliations:** 1 General Surgery, Western Health, Melbourne, AUS; 2 General Surgery, Lyell McEwin Hospital, Adelaide, AUS; 3 Colorectal Surgery, Lyell McEwin Hospital, Adelaide, AUS

**Keywords:** evaluation of clinical assessment in acute diverticulitis, physical signs in acute abdomen, complicated diverticulitis, clinical predictors of complicated diverticulitis, clinical assessment in complicated diverticulitis

## Abstract

Background

Acute diverticulitis is a common surgical condition and one of the leading gastrointestinal conditions that require hospital admission. The presence of complications increases the hospital stay and risk of requiring surgical intervention. This study aimed to investigate the clinical features that can be identified during clinical assessment and evaluate their predictive value and sensitivity in differentiating between complicated and uncomplicated diverticulitis.

Methodology

This retrospective case-control study was performed on patients with acute diverticulitis at Lyell McEwin Hospital, Adelaide, South Australia. Data were collected for patients presenting from January 2015 to December 2017. Patients with acute diverticulitis confirmed by computed tomography (CT) were included in the study. Multiple clinical assessment aspects were reported and compared between complicated diverticulitis and uncomplicated diverticulitis groups.

Results

Data from a total of 116 cases were collected, 10 of which were excluded due to lack of CT diagnosis. Forty-four cases had complicated diverticulitis (case group), and 62 cases had uncomplicated diverticulitis (control group). Twenty-three cases (52.2%) had the first episode of diverticulitis in the complicated group compared to 24 cases (38.7%) in the uncomplicated group, with an odds ratio of 1.73 (0.79-3.789). Eight cases (18.2%) had previously complicated diverticulitis in the complicated group compared to 11 cases (17.7%) in the uncomplicated group, with an odds ratio of 1.03 (0.37-2.82). Six cases (13.6%) had a fever (*T* > 38) in the complicated group compared to two cases (3.2%) in the uncomplicated group, with an odds ratio of 4.74 (0.9-24.7), a sensitivity of only 13.64%, and a specificity of 96.77%. Twelve cases (27.3%) had tachycardia, two cases (4.5%) had hypotension, and five cases (11.4%) had peritonism in the complicated group compared to two cases (3.2%), one case (1.6%), and one case (1.6%) in the uncomplicated group, with odds ratios of 11.25 (2.37-53.4), 2.9 (0.255-33), and 7.82 (0.88-69.5), respectively; sensitivity was 27.27%, 4.55%, and 11.36% for tachycardia, hypotension, and peritonism, whereas specificity was 96.77%, 98.39%, and 98.39%, respectively.

Conclusions

The study found no significant correlation between having complicated diverticulitis and previous episodes of complicated diverticulitis, immunosuppression, pain severity, or change in bowel habits. Perrectal bleeding was found to reduce the risk of having complicated diverticulitis. Our results did not demonstrate a statistically significant relationship between the first episode of diverticulitis and having complicated diverticulitis. Physical signs, when abnormal, are highly specific in predicting complicated diverticulitis. Tachycardia was found to have the highest positive predictive value and odds ratio compared to the other observed physical signs.

## Introduction

Diverticular disease is a common condition that is directly related to the presence of diverticula in the colon wall, whose incidence usually increases with age and is reported to be rising in Western societies [1]. The disease affects approximately 30% of those aged above 60 years and 60% to 80% of those aged above 80 years. Symptoms are thought to develop in 10% to 20% of people with diverticula. The incidence of acute diverticulitis is also increasing, including cases requiring hospital admission [2]. The largest increase (82%) was seen among young people aged 18 to 44 years in one study [3]. Diverticular disease of the colon is a significant cause of hospital admissions and an important contributor to healthcare costs in Western and industrialized societies [4,5]; it is the third most common gastrointestinal illness that requires hospitalization and the leading indication for elective colon resection in the United States [6-8].

There are two recognized classifications of diverticular disease: clinical classification and the Hinchey classification. In clinical classification, diverticular disease is classified as follows: symptomatic uncomplicated disease, recurrent symptomatic disease, and complicated disease. The complicated disease includes hemorrhage, abscess, phlegmon, perforation, purulent and fecal peritonitis, stricture, fistula, and small-bowel obstruction due to postinflammatory adhesions. The modified Hinchey classification describes the clinical stages of perforated diverticular disease, which include stage Ib (pericolic abscess), stage IIa (distant abscess amenable to percutaneous drainage), stage IIb (complex abscess associated with/without fistula), stage III (generalized purulent peritonitis), and stage IV (fecal peritonitis) [9].

There does not seem to be a clear distinction between uncomplicated and complicated diverticulitis in terms of clinical and laboratory findings [9]. Furthermore, some studies found no predictive value of physical signs such as fevers in the diagnosis of complicated diverticulitis [10]. Therefore, this study aims to investigate clinical features that can be identified during clinical assessment and evaluate their predictive value in differentiating between complicated and uncomplicated diverticulitis.

## Materials and methods

This retrospective case-control study was performed for patients with acute diverticulitis at Lyell McEwin Hospital, Adelaide, South Australia. Data were collected for patients admitted to the hospital for inpatient management from January 2015 to December 2017. Patients with the diverticular disease were identified through a diagnosis-specific code used in the hospital. Patients with acute diverticulitis confirmed by computed tomography (CT) were included in the study. Patients with diverticular bleeding, no CT confirmation, and incidental diverticular disease on colonoscopy were excluded.

Patients recruited for the study were divided into two groups. Patients presenting with Hinchey Ia diverticulitis were classified as having *uncomplicated diverticulitis*. Patients who presented with Hinchey Ib, II, III, or IV diverticulitis were classified as having *complicated diverticulitis*. The Hinchey classification was based on radiological reports of CT scans. Patients with complicated diverticulitis (case group) were compared to patients with uncomplicated diverticulitis (control group).

Multiple parameters were collected for each patient in the two study groups: patient demographics, symptoms, number of attacks of diverticulitis, presence of immunosuppression, history of complicated diverticulitis, vital signs, and physical examination and CT findings. These parameters were extracted from patient clinical, laboratory, and radiology records. Pain severity was assessed based on documentation from hospital charts using pain scores of 1 to 10, which relied on subjective self-assessment from the patients when they presented to the emergency department. Immunosuppression was defined as the presence of factors leading to the diminution of host defenses, such as a concurrent history of immunosuppressant medication (e.g., chronic glucocorticoids, azathioprine, cyclosporine, methotrexate, tacrolimus, and antitumor necrosis factor agents), solid organ transplant, extra-colonic active malignant neoplasm, cytotoxic chemotherapy, and congenital or acquired immunodeficiency syndrome.

Binary and continuous data were collected. Continuous variables were either changed to binary data or classified as categorical data that were relevant to clinical practice, including age group categories, blood pressure, heart rate, and temperature. An analysis of binary variables was done using 2 × 2 tables, and odds ratios were calculated with 95% confidence intervals. The chi-square test was used to determine significance. To further investigate the predictive value of the variables that were found to be significant, diagnostic test analyses were used to calculate their sensitivity, specificity, positive predictive value, and negative predictive value, which were calculated using the prevalence of complicated diverticulitis in the study population.

## Results

Data from a total of 116 cases were collected, of which 10 were excluded due to missing CT diagnosis data. Forty-four cases with complicated diverticulitis served as the case group, and 62 cases of uncomplicated diverticulitis served as the control group. The two groups were comparable in demographics and other factors that were expected to be relevant in determining outcomes (Table 1).

**Table 1:** Characteristics of the study groups.

	Complicated (case) group	Uncomplicated (control) group
Frequency	44	62
Sex, *n* (%)		
Male	26 (59)	32 (52)
Female	18 (41)	30 (48)
Age (years), mean (SD)	56.7 (17)	56.2 (14.6)
First episode, *n* (%)	23 (52.3)	24 (38.7)
Recurrent episode, *n* (%)	21 (47.7)	38 (61)
Immunosuppression, *n* (%)	3 (6.8)	5 (8)
Previous episodes of complicated diverticulitis, *n* (%)	8 (18.2)	11 (17.8)

Different aspects of clinical assessment were categorized and reported in a standard clinical assessment manner (i.e., history, symptoms, and signs; Table 2).

**Table 2:** Study group comparisons. CI, confidence interval; PR, perrectal; HR, heart rate; SBP, systolic blood pressure

	Complicated (case) group, *n *(%)	Uncomplicated (control) group, *n *(%)	Odds ratio (95% CI)
History			
First episode	23 (52.3)	24 (38.7)	1.734 (0.794-3.789)
Previous episodes of complicated diverticulitis	8 (18.2)	11 (17.8)	1.03 (0.377-2.82)
Immunosuppression	3 (6.8)	5 (8)	0.83 (0.188-3.69)
Symptoms			
Pain			
Mild	20 (45.5)	14 (22.6)	
Moderate	13 (29.5)	24 (38.7)	
Severe	11 (25)	24 (38.7)	
Diarrhea	4 (9)	11 (17.7)	0.463 (0.137-1.565)
Constipation	15 (34)	16 (25.8)	1.488 (0.64-3.46)
PR bleeding	1 (2.2)	10 (16.1)	0.12 (0.015-0.98)
Signs			
Fever	6 (13.6)	2 (3.2)	4.737 (0.9-24.7)
Tachycardia (HR > 100)	12 (27.3)	2 (3.2)	11.25 (2.37-53.4)
Hypotension (SBP < 100)	2 (4.5)	1 (1.6)	2.9 (0.255-33)
Peritonism	5 (11.4)	1 (1.6)	7.82 (0.88-69.5)

There were 23 cases (52.2%) with the first episode of diverticulitis in the complicated group compared to 24 (38.7%) cases in the uncomplicated group, with an odds ratio of 1.73 (0.79-3.789), which did not reach statistical significance. Eight cases (18.2%) had previous episodes of complicated diverticulitis in the complicated group compared to 11 (17.7%) cases in the uncomplicated group, with an odds ratio of 1.03 (0.37-2.82).

Common symptoms of diverticulitis are reported in the symptoms category given in Table 2. The pain was reported in three grades, namely, mild, moderate, and severe, which were subjectively recorded by the patients upon presenting to the emergency department. In the complicated group, the pain was mild in 20 cases (45.5%), moderate in 13 cases (29.5%), and severe in 11 cases (25%), compared to 14 cases (22.6%), 24 cases (38.7%), and 24 cases (38.7%) in the control group, respectively. There was one case (2.2%) with perrectal (PR) bleeding in the complicated group compared to 10 cases (16.1%) in the control group, with an odds ratio of 0.12 (0.015-0.98).

Relevant physical examination signs are reported and compared in the signs category given in Table 2. There were six cases (13.6%) with fever (*T* > 38) in the complicated group compared to two cases (3.2%) in the uncomplicated group, with an odds ratio of 4.74 (0.9-24.7). There were 12 cases (27.3%) with tachycardia, two cases (4.5%) with hypotension, and five cases (11.4%) with peritonism in the complicated group compared to two cases (3.2%), one case (1.6%), and one case (1.6%) in the uncomplicated group, respectively. The corresponding odds ratios were as follows: 11.25 (2.37-53.4) for tachycardia, 2.9 (0.255-33) for hypotension, and 7.82 (0.88-69.5) for peritonism.

As there was a correlation between physical examination signs and the risk of diverticulitis, the sensitivity and specificity of each of the studied signs were calculated, as shown in Table 3. The highest sensitivity was reported for tachycardia, but even then, this value was only 27.27%. Similarly, sensitivity was found to be low for the other physical signs, with 13.64% for fever, 4.55% for hypotension, and 11.36% for peritonism. In contrast, specificity was high for all physical signs, with 96.8% for fever and tachycardia and 98.4% for hypotension and peritonism. Positive predictive values were highest in tachycardia and peritonism, with values of 67.9% and 63.8%, respectively.

**Table 3:** Physical examination signs diagnostic evaluation. PPV, positive predictive value; NPV, negative predictive value

Signs	Sensitivity	Specificity	PPV	NPV
Fever	13.64	96.77	51.38	81.76
Tachycardia	27.27	96.77	67.88	84.18
Hypotension	4.55	98.39	41.33	80.48
Peritonism	11.36	98.39	63.79	81.62

## Discussion

As clinical assessment is an important part of any patient surgical management, it is crucial to evaluate its accuracy in detecting cases of complicated diverticulitis and determining the aspects that have a higher weight and are more significant predictors of complicated diverticulitis. We found that the first presentation of diverticulitis has an increased risk of having complicated diverticulitis, with an odds ratio of 1.74. However, this correlation failed to achieve statistical significance, likely due to the sample size of our study.

PR bleeding was found to reduce the risk of complicated diverticulitis. This correlation was statistically significant within our study population. All studied physical signs were found to increase the risk of complicated diverticulitis when abnormal. In this study, statistical significance was only demonstrated for tachycardia. Having a heart rate above 100 beats per minute (bpm) had the highest significance in predicting complicated diverticulitis compared to the other studied variables, with an odds ratio of 11.25 (2.37-53.4).

We also investigated the diagnostic value of the presence of abnormal physical signs in predicting complicated diverticulitis. We found that although there was a significant increase in risk with abnormal clinical signs, their sensitivity as predictors of complicated diverticulitis was low. The highest sensitivity was 27.3% (14.96%-42.79%) for tachycardia, with a positive predictive value of 67.8%, which correlated to a high number of cases of complicated diverticulitis that would be missed if depending solely on clinical assessment. In contrast, the specificity of these signs when abnormal was very high, at 96% for fever and tachycardia and 98% for hypotension and peritonism. We found no significant correlation between having complicated diverticulitis and previous episodes of complicated diverticulitis, immunosuppression, pain severity, or changes in bowel habits.

The study was mainly limited in its sample size. A larger study population might be required to demonstrate statistical significance in some of the important correlations found in the study.

## Conclusions

Our results showed that having a first episode of acute diverticulitis was found to correlate with an increased risk of complicated diverticulitis, but the correlation was not statistically significant due to the small sample size. PR bleeding was associated with a lower risk of complicated diverticulitis, whereas no significant correlation was found between complicated diverticulitis and previous episodes of the disease, immunosuppression, pain severity, or changes in bowel habits.

Although physical examination signs are important in the clinical assessment of acute diverticulitis, we found that they lacked sensitivity in predicting cases of complicated diverticulitis. Therefore, we predict a high number of undiagnosed cases of complicated diverticulitis if depending on clinical assessment alone. In contrast, physical signs, when abnormal, were found to be highly specific in predicting complicated diverticulitis. Future studies should further investigate biochemical markers and their diagnostic merit to determine which aspects of assessment have the highest value in predicting complicated diverticulitis and can be implemented in clinical practice. 
